# Ecological Change: Life Lessons

**Published:** 2005-12

**Authors:** Dorothy Bonn

“All global environmental change eventually ends up as a human health problem,” said Eric Chivian, director of the Harvard Center for Health and the Global Environment, opening the August 2005 First International Conference on Health and Biodiversity in Galway, Ireland. Speaker after speaker showed how careless disregard for the environment and its variety of life forms squanders potential new medicines, endangers our food security, and exposes us to new risks of infectious disease.

Many frequently prescribed drugs are derived from or patterned after compounds in natural sources, Chivian noted. For example, ziconotide—a pain killer 1,000 times more powerful than morphine—comes from marine cone snails that inhabit narrow ranges in coral reefs and thus are increasingly endangered by coral bleaching, mostly from global warming. How many other useful species are lost without our ever recognizing their potential?

Species loss may also mean the loss of valuable models for medical research, said Chivian. Black bears, which hibernate for several months over the winter without losing bone mass, could provide a clue to the cause of osteoporosis, an enormous public health problem. But bear populations in many parts of the world are threatened by habitat destruction and overhunting.

Discussion of sustainable food systems for developing countries focused on promoting the use of indigenous plants. In Lebanon, where diets are high in bread and refined grains but low in fruits, vegetables, and fish, a quarter of the children are overweight and a third of the women of child-bearing age are anemic. Malek Batal, a nutrition professor at the American University of Beirut, is exploring how wild plants such as fennel, mint, and salsify have the potential to increase diversity of nutrient intake and food security in poor communities. He found that wild plants offer antioxidants, flavonoids, fiber, iron, calcium, and many other nutrients. Being easily accessible, easy to use, and palatable, they also contribute to food security.

Interfering with ecosystems can have dire consequences for biodiversity, as conservation biologist Diana Bell of the University of East Anglia explained: when the South American myxoma virus was introduced into Europe in the 1950s to control rabbit populations, it contributed to the collapse of a species-rich ecosystem in which the rabbit was the keystone prey for more than 45 predators. Bell also identified the illegal trade in wildlife (especially small carnivores) in Southeast Asia as a dual threat to human health (as the origin of the SARS coronavirus) and massive species loss in this “biodiversity hot spot.” She believes an interdisciplinary approach involving ecologists, microbiologists, medical specialists, and others will best advance research in the twin fields of human health and species loss.

The time to address biodiversity loss is now, speakers agreed. As Chivian said, “We are in deep, deep trouble with what we are doing to life on Earth. . . . We are tampering with the life support systems of the Earth in ways that we barely understand.”

